# How Morphology Shapes Survival in Invasive Squamous Cell Carcinoma of the Lung

**DOI:** 10.3390/diagnostics14202264

**Published:** 2024-10-11

**Authors:** Angela-Ștefania Marghescu, Silviu Vlăsceanu, Mădălina Preda, Beatrice Mahler, Ioana Anca Bădărău, Loredana Sabina Cornelia Manolescu, Mirela Țigău, Cristina Teleagă, Corina Elena Toader, Alexandru Daniel Radu, Alexandru Stoichiță, Mariana Costache

**Affiliations:** 1 Pathological Anatomy Discipline, Faculty of Medicine, Carol Davila University of Medicine and Pharmacy, 020021 Bucharest, Romania; angela.varban@drd.umfcd.ro (A.-Ș.M.); mariana.costache@umfcd.ro (M.C.); 2 Department of Research, Marius Nasta Institute of Pneumophthisiology, 050159 Bucharest, Romania; mirela.tigau@marius-nasta.ro (M.Ț.); cristina.teleaga@marius-nasta.ro (C.T.); alexandru.radu@marius-nasta.ro (A.D.R.); alexandru.stoichita@drd.umfcd.ro (A.S.); 3 Physiology III Discipline, Faculty of Medicine, Carol Davila University of Medicine and Pharmacy, 020021 Bucharest, Romania; anca.badarau@umfcd.ro; 4 Department of Thoracic Surgery, Marius Nasta Institute of Pneumophthisiology, 050159 Bucharest, Romania; 5 Department of Microbiology, Parasitology and Virology, Faculty of Midwives and Nursing, Carol Davila University of Medicine and Pharmacy, 020021 Bucharest, Romania; loredana.manolescu@umfcd.ro; 6 Clinical Laboratory of Medical Microbiology, Marius Nasta Institute of Pneumology, 050159 Bucharest, Romania; 7 Pneumoftisiology II Discipline, Faculty of Medicine, Carol Davila University of Medicine and Pharmacy, 020021 Bucharest, Romania; beatrice.mahler@umfcd.ro; 8 Department of Pulmonology, Marius Nasta Institute of Pneumophysiology, 050159 Bucharest, Romania; 9 Pathology Department, Marius Nasta Institute of Pneumophthisiology, 050159 Bucharest, Romania; toader.corina@marius-nasta.ro; 10 Pathology Department, University Emergency Hospital Bucharest, 050098 Bucharest, Romania

**Keywords:** squamous cell carcinoma, lung, survival, prognostic, morphological factors, risk stratification, grading

## Abstract

Background and Objectives: Squamous cell carcinoma (SQCC) represents a significant proportion of human malignancies affecting various anatomical sites, including the lung. Understanding the prognostic factors is crucial for establishing effective risk stratification in these patients, as multiple critical aspects significantly impact overall survival. Materials and Methods: A retrospective study was conducted on 99 patients with operable lung SQCC treated at a tertiary center. The exclusion criteria included patients under 18, those with in situ or metastatic SQCC, and those who received neoadjuvant therapy. The surgical specimens were re-analyzed, and data were collected on multiple variables, including pTNM staging, tumor characteristics, and overall survival (OS). The Kaplan–Meier survival analysis and Cox regression models were used to identify significant prognostic factors. Results: The Kaplan–Meier analysis showed a median survival of 36 months with a 65.65% mortality rate. Significant factors influencing survival included keratinization, histological grading, tumor size and stage, pleural invasion, tumor cell arrangement, tumor budding, spread through air space (STAS), and mitotic index. A multiple Cox regression highlighted the nonkeratinizing tumors, advanced pT stages, single-cell invasion, and high mitotic index as key predictors of poorer outcomes. The nonkeratinizing tumors showed higher mortality and shorter median survival rates compared to keratinizing tumors. The tumor staging, cell arrangement, and tumor budding significantly impacted the survival curves. Conclusions: The study underscores the importance of detailed histopathological evaluations in lung SQCC. The nonkeratinizing tumors, advanced pT stage, single-cell invasion, and high mitotic index were associated with higher hazard rates, emphasizing the need for a comprehensive grading system incorporating these factors to improve prognostic accuracy and guide treatment strategies.

## 1. Introduction

Squamous cell carcinoma (SQCC) is one of the most prevalent types of human malignancy, characterized by its epithelial origin and a wide range of potential sites, including the lung, uterine cervix, skin, oral cavity, esophagus, anus, larynx, and penis, among others. It can arise from any squamous epithelium, including areas of metaplasia, and is associated with high mortality. While the morphological characteristics of SQCC are similar, the origin of the carcinoma significantly influences its behavior, clinical presentation, and treatment [1,2,3,4]. Besides SQCC, other types of cancers that can affect the lungs include adenocarcinoma, large cell neuroendocrine carcinoma, small cell neuroendocrine carcinoma, carcinoids, pleomorphic carcinoma, lymphoma, sarcoma, and metastatic cancers from other parts of the body [1].

Pathological reports for these tumors typically include pTNM staging, histological grading, assessments of lymphovascular and perineural invasion, and evaluation of resection margins. Regardless of the origin, determining the degree of differentiation in SQCC remains a complex task for pathologists due to the lack of validated grading systems or criteria. The accurate evaluation of these factors is crucial for risk stratification, prognosis, and therapeutic management of each case [1,4,5].

What is known is that nuclear pleomorphism and keratin synthesis are the two most analyzed parameters when grading SQCC. However, there remains a lack of standardization in this grading process, unlike in lung adenocarcinoma, where architectural patterns are strictly quantified. These aspects are not yet sufficiently described for lung SQCC. We believe that a thorough analysis of the morphological characteristics in SQCC is essential, as these factors could significantly influence patient prognosis. Integrating these characteristics into a new grading system may be beneficial [6].

The study aims to determine if pathological factors (e.g., tumor size and stage, keratinization, tumor cell arrangement, nuclear pleomorphism, mitosis, tumor budding, spread through air space—STAS) independently predict clinical outcomes, specifically overall survival (OS).

## 2. Materials and Methods

We conducted a retrospective, observational, longitudinal, cross-sectional, non-randomized study on a cohort of 99 patients with operable stages of lung cancer.

The inclusion criteria for this study were established to ensure a clinically relevant participant population. Specifically, individuals aged 18 years or older diagnosed with invasive squamous cell carcinoma of the lung were considered eligible for inclusion. The participants were required to have undergone surgical intervention, which could include pneumonectomy, bilobectomy, or lobectomy, all performed with the intent of achieving curative outcomes. The recruitment of subjects took place exclusively at the Marius Nasta Pneumophtisiology Institute during the year 2016. Additionally, it was mandated that all prospective participants provide informed consent before their inclusion in the study, thereby upholding ethical standards in research.

All patients received the best possible therapy evaluation, with surgical interventions carried out in compliance with globally recognized guidelines.

The surgical specimens were processed using standard methods, fixed in 10% buffered formalin, and stained with hematoxylin-eosin.

The exclusion criteria were as follows: patients under 18 years of age, those with in situ or metastatic squamous cell carcinoma, cases diagnosed exclusively via Endobronchial Ultrasound (EBUS) or biopsy specimens, patients who received neoadjuvant therapy, and those with diagnoses or surgeries outside the specified timeframe or at another medical unit.

The histologic slides were retrieved from the Pathology Department archive and reanalyzed using a BX46 Olympus microscope (Olympus Corporation, Tokyo, Japan). Epidemiological, clinical, and paraclinical data were collected from the institute’s database and centralized in Microsoft Excel. The variables evaluated included pTNM staging; lymphovascular, perineural, and pleural invasion; resection margins; degree of keratinization; necrosis; tumoral budding; STAS; mitotic index; desmoplasia; neoplastic cell arrangement in the central and peripheral part of the tumor; nuclear pleomorphism; nucleoli appearance; histological grade; tumor location and size; and overall survival. The survival status was also documented.

Many of the analyzed parameters are subjective and challenging to quantify due to the lack of international guidelines for this evaluation.

**Figure 1:**
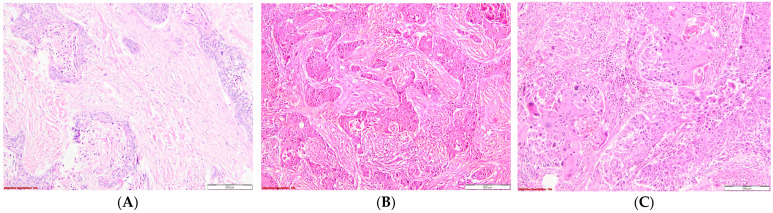
Evaluation of the stromal desmoplasia in SQCC: (**A**). Severe stromal desmoplasia characterized by abundant keloid-like collagen deposition surrounding the tumor nests; HE, 100×; (**B**). Moderate stromal desmoplasia, with moderate collagen buildup in the tumoral interstitium; HE, 100×; (**C**). Mild stromal desmoplasia, with barely perceptible interstitial collagen accumulation; HE, 100×.

The evaluation of stromal desmoplasia was conducted according to the accompanying images (Figure 1).

**Figure 2:**
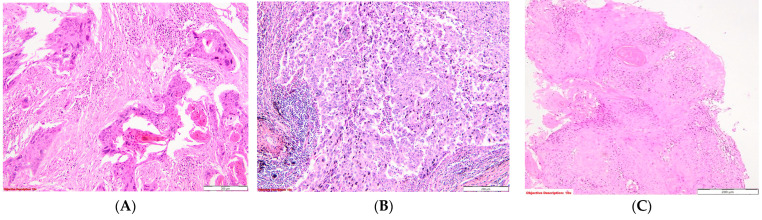
Evaluation of the nuclear pleomorphism in SQCC: (**A**). Severe nuclear pleomorphism is characterized by tumor cells exhibiting large, intensely hyperchromatic nuclei with pronounced variation in size and shape.; HE, 100×; (**B**). Moderate nuclear pleomorphism in tumor cells involves noticeable but not extreme variability in the size and shape of the nuclei; HE, 100×; and (**C**). Mild nuclear pleomorphism—minor differences between normal and malignant cells’ nuclear abnormalities; HE, 100×.

The methodology for evaluating nuclear pleomorphism adhered closely to the aspects depicted in the accompanying images (Figure 2).

The assessment of tumor cell arrangement included the categorization into large nests (≥5 tumor cells), small nests (2–4 tumor cells), and single-cell invasion. The mitotic index was classified as either low (<15 mitosis/10 high power field (HPF)) or high (≥15 mitosis/10 HPF).

Tumor budding refers to the presence of small clusters (<5 tumor cells) or single cells of cancer cells at the invasive front of a tumor.

Images were captured using a digital camera from an Olympus SC50 microscope (Olympus Corporation, Tokyo, Japan). A statistical analysis was performed using R software, version 4.3.3 (2024, The R Foundation for Statistical Computing) with additional packages including survival [7], superminer [8], and gtsummary [9] (accessed on 1 June 2024).

The principal endpoint was overall survival (OS), defined as the time from surgical intervention to patient death or 1 March 2023, quantified using the restricted mean survival time (RMST) and median survival.

The Kaplan–Meier survival analysis was performed on the entire cohort and various categorical strata. The Cox regression (both simple and multiple) identified the evaluated parameters, which served as hazard factors for patient outcomes.

This analysis could potentially apply to squamous cell carcinomas originating in other organs; however, further studies are necessary to confirm this.

## 3. Results

**Figure 3:**
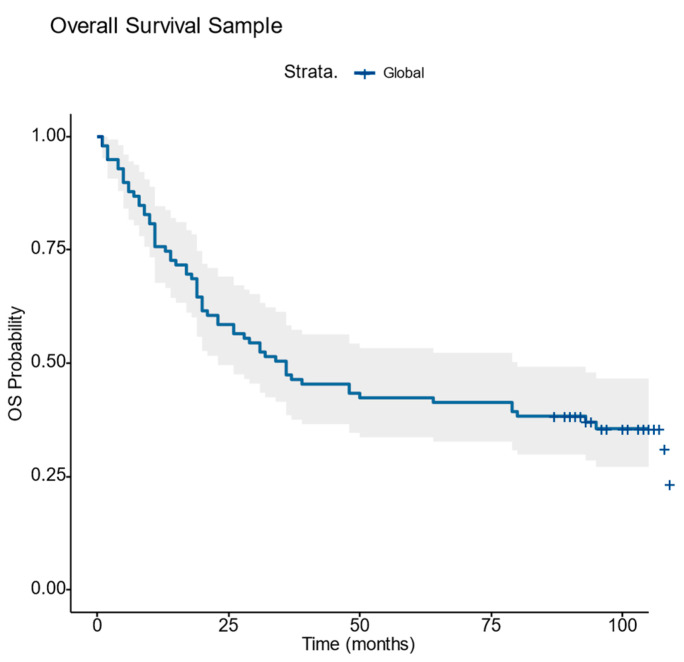
Overall survival Graph.

The Kaplan–Meier survival analysis was conducted on the entire cohort (N = 99 patients), of which 60% were men. The median age of the patients was 63 years old. Figure 3 and Table 1 depict the overall survival outcomes revealing a median survival of 36 months and a mortality rate of 65.65%.

In Table 2, we conducted an analysis of several parameters including age; desmoplasia, necrosis; keratinization; histological grading and staging; tumor size; lymphovascular, perineural, and pleural invasion; tumor budding; STAS; prominence of nucleoli; nuclear pleomorphism; mitotic index; neoplastic cell arrangement in the central and peripheral parts of the tumor and resection margins; and their correlation with survival.

Several factors significantly influenced the risk of death in lung squamous cell carcinoma. A lack of keratinization increased the hazard rate (HR) by 2.18 times compared to the keratinizing tumors. G3 tumors are associated with a 2.53-fold increase in the hazard rate compared to G1 tumors. The tumor size was also evaluated, each 10 mm increase was linked to a 14% rise in the hazard rate. Analyzing the pT stage, the hazard rate increased 3.45 times for pT2 and nearly five times for pT3 compared to pT1.

**Figure 4:**
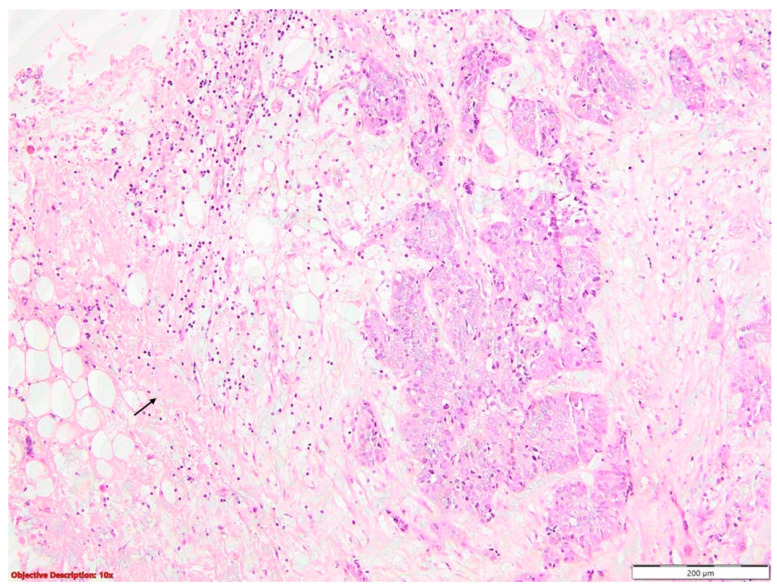
Infiltration of the parietal pleura (→) by nests of squamous cell carcinoma, characterized by large neoplastic cells with vesicular nuclei and eosinophilic cytoplasm (PL3); HE, 100×.

Pleural invasion (PL) beyond the external lamina elastica (PL2/PL3) doubled the hazard rate compared to PL0/PL1 (Figure 4).

**Figure 5:**
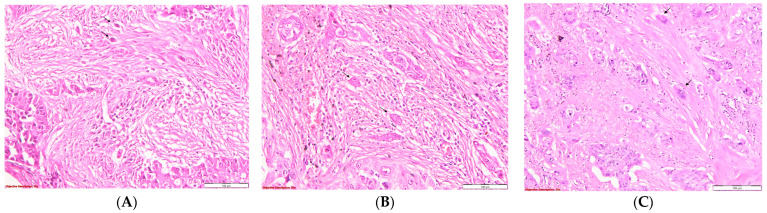
Neoplastic cell arrangement in the central part of the tumor: (**A**). Single-cell invasion: isolated neoplastic cells (→) infiltrating the keloid-like tumoral stroma; HE, 200×; (**B**). Small nests: groups of 2–4 neoplastic cells (→) invading the tumoral stroma; HE, 200×; and (**C**). Large nests: groups of more than 5 tumor cells (→) infiltrating the collagenous stroma of the tumor; HE, 200×.

The disposition of tumor cells in the central part of proliferation (Figure 5) also affected the risk, with single-cell invasion increasing the hazard rate by 6.58 times and small-nest disposition increasing it by 2.46 times.

**Figure 6:**
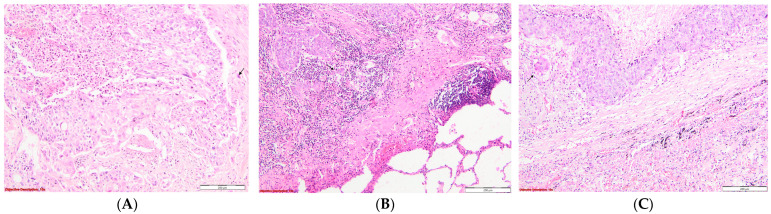
Neoplastic cell arrangement in the peripheral part of the tumor: (**A**). Single-cell invasion: solitary cancerous cells (→) at the tumor periphery, HE, 100×; (**B**). Small nests: 2–4 tumor cells (→) infiltrating at the lung tissue-tumor junction, HE, 100×; and (**C**). Large nests: ≥5 tumor cells (→) at the peripheral part of the tumor proliferation, HE, 100×.

In the periphery of proliferation, single-cell invasion raised the hazard rate by 2.82 times compared to the large-nest disposition (Figure 6).

**Figure 7:**
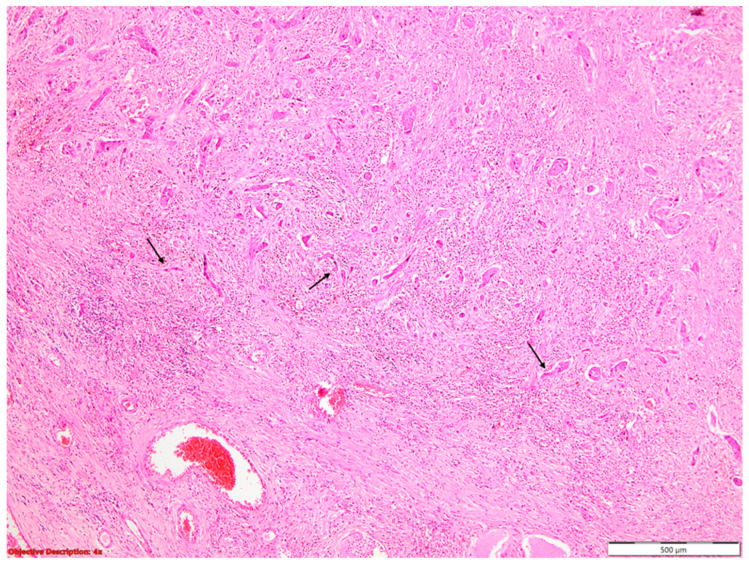
Tumoral budding in SQCC—small clusters or individual neoplastic cells (→) that are evident at the invasive front of a tumor; HE, 40×.

**Figure 8:**
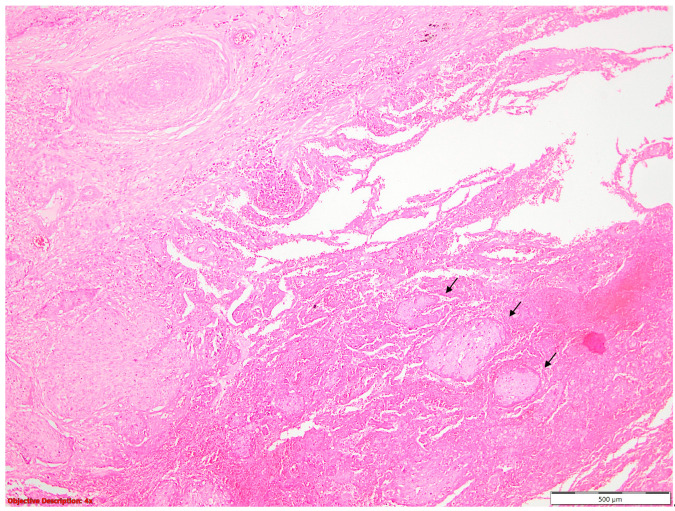
STAS in lung SQCC: Spread Through Air Space is evident as clusters of neoplastic cells (→) within the alveoli, located at a distance from the primary tumor; HE, 40×.

The presence of tumor budding (Figure 7) nearly tripled the hazard rate, while the STAS (Figure 8) increased it by approximately two times.

**Figure 9:**
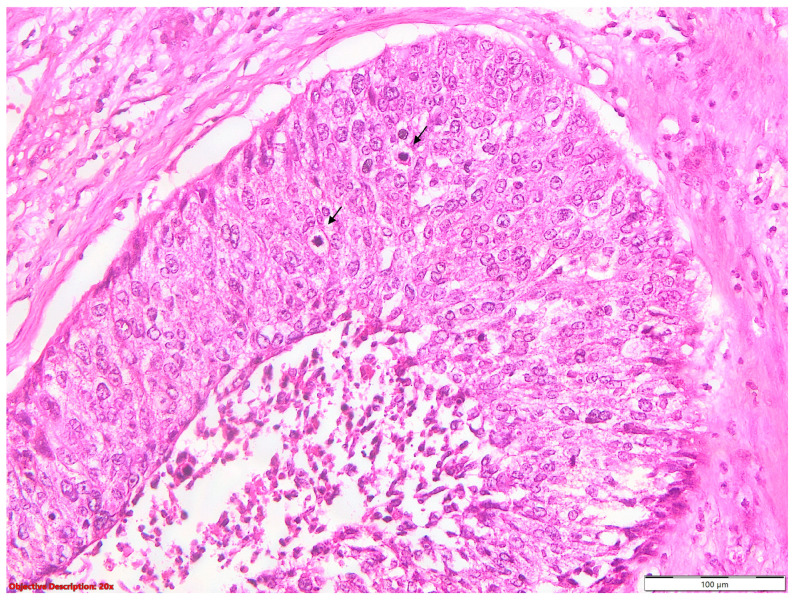
Mitotic activity in SQCC: tumoral cells have vesicular, moderate pleomorphic nuclei, with evident nucleoli, and two obvious atypical mitoses in the field (→); HE, 200×.

Additionally, a high mitotic index determined a 1.6-fold increase in the hazard rate, compared to a low mitotic index (Figure 9).

These factors highlight the importance of detailed histopathological evaluation in determining the prognosis and guiding treatment strategies for lung squamous cell carcinoma.

The predictors, which had a significant influence on statistics, were used in a multiple Cox regression, the initial model is illustrated below (Table 3).

Using a retrograde selection algorithm (backward selection), we built a model with all the predictors having *p*-values below 0.10 (Table 4).

Multiple factors influenced the risk of death in lung squamous cell carcinoma.

The nonkeratinizing tumors increased the hazard rate by 1.81 times compared to the keratinizing tumors. The pT stage was crucial, with stage T2 increasing the hazard rate by 3.65 times and stage T3 by 4.14 times compared to T1. The central disposition of tumoral cells significantly impacted the risk, with single-cell invasion increasing the hazard rate by 4.27 times and the small-nest disposition by two times compared to the large-nest arrangement. Tumor budding also elevated the hazard rate by 1.66 times. Additionally, a high mitotic index nearly doubled the hazard rate compared to a low mitotic index.

**Figure 10:**
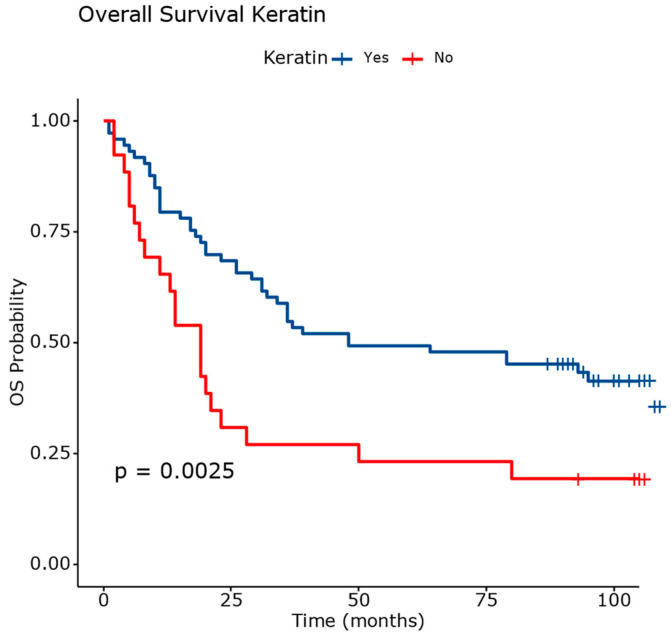
Overall survival analysis based on evaluation of tumor cell keratin synthesis. The differences between survival curves were statistically significant.

**Figure 11:**
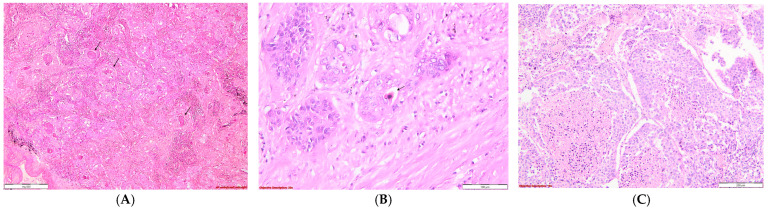
Squamous cell carcinoma: *Keratinizing type* (**A**,**B**): (**A**). Tumoral nests, some exhibiting keratin pearls (→) formation in their center, HE, 40×; (**B**). A lower degree of keratinization is observed, with only diskeratocytes (→) present, characterized by intense eosinophilic cytoplasm and altered nuclei, HE, 200×; and *Nonkeratinizing type* (**C**): Poorly differentiated proliferation, composed of large tumoral cells arranged in nests, without keratinization, and exhibiting important necrosis, HE, 100×.

The data from Figure 10 and Table 5 highlight significant differences in overall survival outcomes between the nonkeratinizing and keratinizing squamous cell carcinomas. The nonkeratinizing tumors (Figure 11) exhibited a higher mortality rate of 84.61% and a longer median survival of 48 months compared to keratinizing tumors (Figure 11), which had a mortality rate of 58.90% and a shorter median survival of 19 months. These findings underscore the prognostic significance of tumor cell keratin synthesis as a determinant of patient outcomes in squamous cell carcinoma.

**Figure 12:**
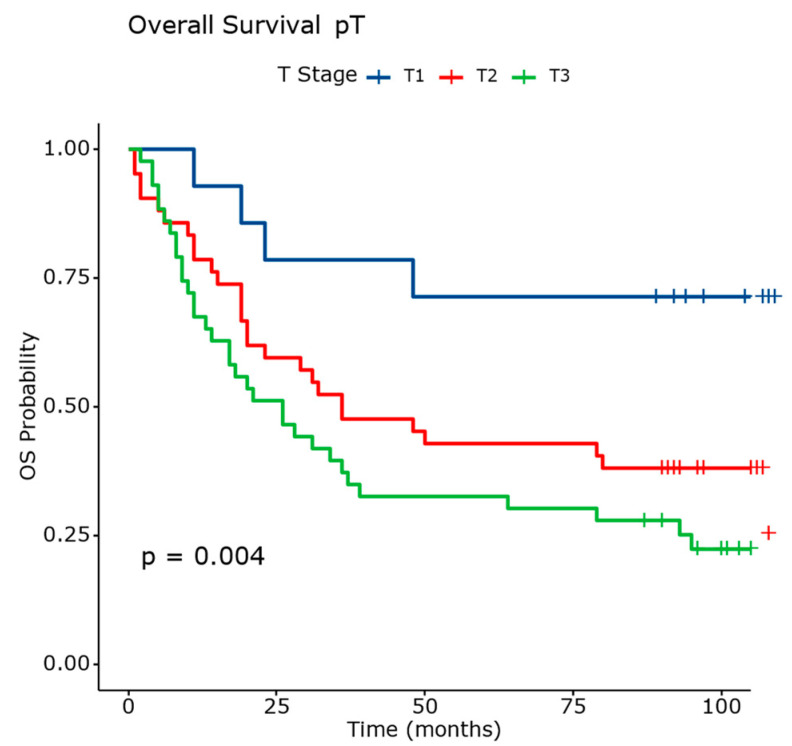
Overall survival analysis based on evaluation of pT. The differences between survival curves were statistically significant.

Figure 12 and Table 6 present comprehensive data on the overall survival outcomes stratified by pT stage in patients with squamous cell carcinoma. The pT1 stage exhibits a lower mortality rate of 28.57% and an RMST of 85.10. The patients with pT2 tumors show a higher mortality rate of 64.28% and an RMST of 56.10. The median survival is 36 months (95% CI: 20.00 to N/A). The pT3 and pT4 are advanced stages associated with higher mortality rates (79.06%) and lower RMST (44.00). The median survival ranges from 26 months (95% CI: 14.00 to 64.00).

These findings underscore the prognostic significance of pT staging in predicting the survival outcomes in squamous cell carcinoma. They emphasize the importance of accurate staging for guiding treatment decisions and prognosticating patient outcomes.

**Figure 13:**
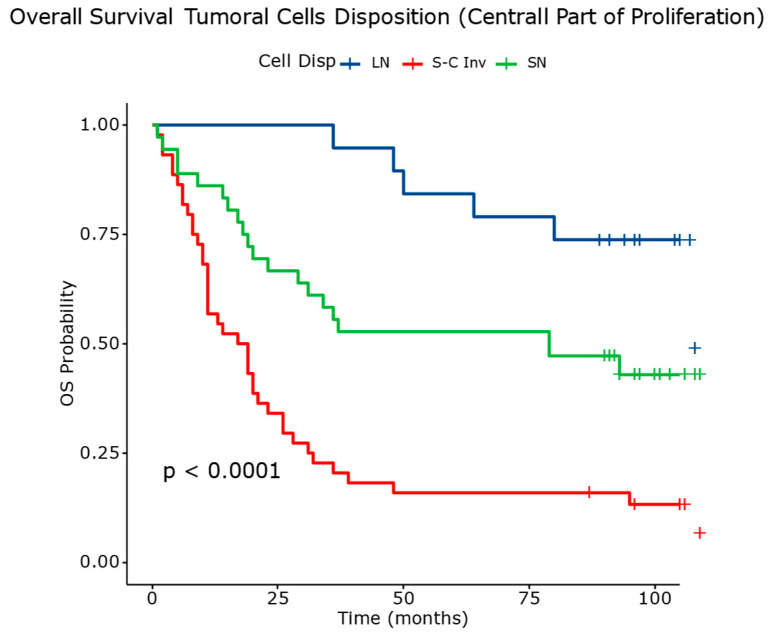
Overall survival analysis based on evaluation of neoplastic cells arrangement in the central part of the tumor.

Table 7 and Figure 13 depict the overall survival outcomes stratified by the arrangement of neoplastic cells in the central part of the tumor. The patients with tumors characterized by large nests had a lower mortality rate (31.57%) and longer median survival of 108 months. The patients with tumors exhibiting single-cell invasion had a significantly higher mortality rate (88.63%) and shorter median survival of 18 months. The tumors with small nests showed an intermediate mortality rate (57.14%) and a median survival of 79 months.

The differences observed in the survival curves between these groups were statistically significant, emphasizing the prognostic significance of neoplastic cell arrangement in the central part of the tumor. These findings underscore the importance of histological characterization in predicting outcomes and guiding treatment strategies for patients with squamous cell carcinoma.

**Figure 14:**
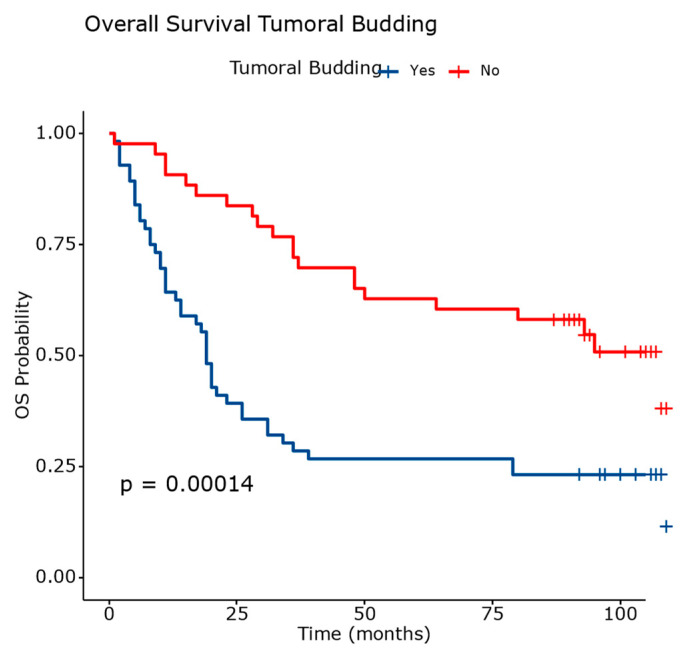
Overall survival analysis based on evaluation of tumor budding. The differences between survival curves were statistically significant.

Figure 14 and Table 8 present the overall survival outcomes stratified by the presence or absence of tumor budding in patients with squamous cell carcinoma.

Patients with tumor budding have a higher mortality rate of 78.57% and a shorter RMST of 38.90. The median survival is 19 months (95% CI: 14.00 to 21.00).

Patients without tumor budding exhibit a lower mortality rate of 48.83% and a longer RMST of 75.50. The median survival is 108 months (95% CI: 50.00 to N/A).

These results indicate that the presence of tumor budding is associated with poorer overall survival outcomes in squamous cell carcinoma. The significant differences observed in the survival curves between the two groups underscore the prognostic relevance of tumor budding as a potential biomarker for predicting patient outcomes.

**Figure 15:**
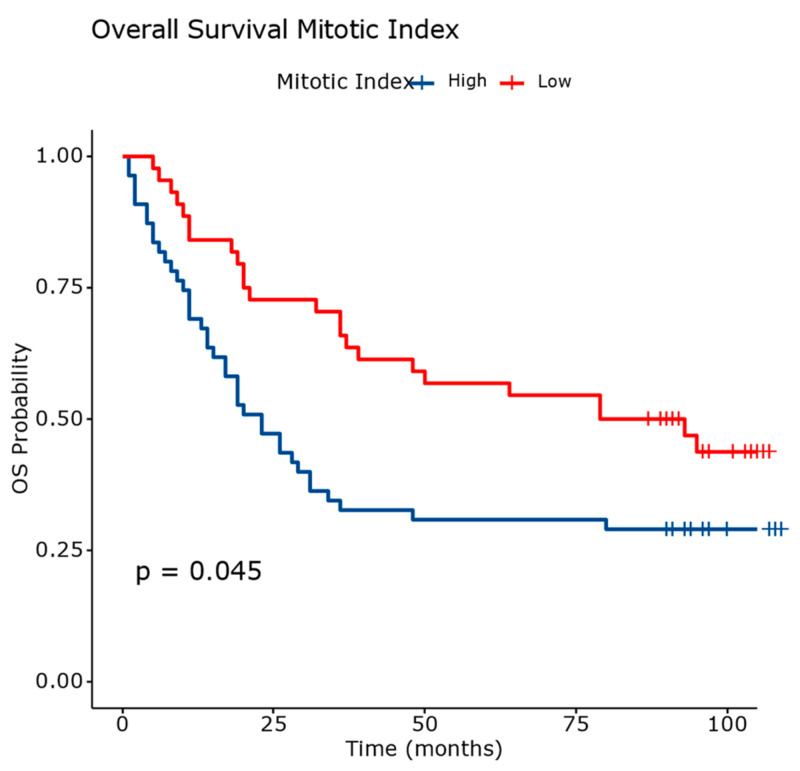
Overall survival analysis based on evaluation of the mitotic index. The differences between survival curves were statistically significant.

Figure 15 and Table 9 present comprehensive data on overall survival outcomes stratified by the mitotic index in patients with squamous cell carcinoma.

Patients with a high mitotic index exhibit a mortality rate of 70.90%, with a restricted mean survival time (RMST) of 44.10 months. The median survival is 23 months (95% CI: 17.00 to 24.00).

Patients with a low mitotic index show a lower mortality rate of 59.09%. They have a longer RMST of 68.30 months and a significantly longer median survival of 86 months (95% CI: 39.00 to N/A).

These results underscore the prognostic significance of the mitotic index in predicting survival outcomes in squamous cell carcinoma. The differences observed in survival between high and low mitotic index groups highlight the potential utility of this parameter in clinical decision-making and patient management.

## 4. Discussion

Our research contributes significantly to the understanding of prognostic factors in lung squamous cell carcinoma, shedding light on several critical aspects that influence overall survival. Among these factors, the neoplastic cell arrangement, tumor budding, mitotic index, keratinization, and tumor staging emerge as key determinants. These findings underscore the complex interplay between the histological characteristics and clinical outcomes in SQCC.

Besides the pathological stage, single-cell invasion and tumor budding are the most frequently mentioned in the medical literature as significant independent prognostic factors for overall survival in patients with lung squamous cell carcinomas [10,11,12,13,14,15,16,17,18,19,20].

Notably, our study aligns with previous literature emphasizing the pivotal roles of single-cell invasion and tumor budding as independent prognostic markers beyond pathological staging [1,2,3,4,5]. These factors are consistently highlighted for their predictive value across diverse patient populations, underscoring their significance in clinical decision-making not only in lung cancer but also in other malignancies (such as uterine cervix) [21,22].

However, challenges persist with the current grading systems. Some research highlights the shortcomings of the current WHO (World Health Organisation) grading system for squamous cell carcinomas across various anatomical sites, emphasizing its limited prognostic power and substantial interobserver variability. M. Jesinghaus et al., proposing an alternative grading approach termed cellular dissociation grading (CDG), which integrates quantitative measures like tumor budding and qualitative assessments such as cell nest size, offer a promising alternative [12].

The study suggests that the CDG provides more reliable prognostic information compared to the traditional WHO system. Its application in both surgical specimens and biopsy settings allows for a robust outcome prediction in both resectable and non-resectable cases [12].

Regarding the histological subtyping (keratinizing, nonkeratinizing, basaloid), divergent findings exist in the literature. Some studies suggest that they have no prognostic significance [10,23], while others argue that keratinization is linked to poorer prognosis and overall survival [11,22]. Other analyses revealed improved survival in the basaloid subtype [24].

This discrepancy underscores the complexity of histological subtypes and their varying impacts on clinical outcomes, necessitating further investigation and validation.

Further validating our findings, N. Zombori-Tóth et al. highlight the prognostic relevance of tumor budding, minimal cell nest size, and STAS in SQCC. These factors contribute crucial insights for stratifying patients based on prognosis, highlighting their utility in clinical practice. Single-cell invasion correlated significantly with a higher nodal involvement and stage. Tumor budding was associated with a higher cancer stage. Tumor budding was most frequently observed in the keratinizing histological subtype of SQCC and was linked to infiltrative tumor borders, smaller minimal cell nest sizes, single-cell invasion, and various forms of invasion (pleural, vascular, lymphovascular) [13].

C. Neppl et al. performed a study focused on the same parameters as N. Zombori-Tóth et al.’s; the difference between the two studies regards the STAS, which did not prove a significant prognostic value in C. Neppl et al.’s research [13,14].

Other studies mentioned that while the STAS is a critical prognostic factor in both lung SQCC and adenocarcinoma, there are notable differences in its prevalence, morphological presentation, and association with tumor subtypes. In SQCC the STAS appears uniformly as solid tumor nests across different histological subtypes, while in adenocarcinoma it presents in various forms (micropapillary clusters, solid nests, or single cells) and is more common in aggressive subtypes. Despite these differences, in both types of lung cancer the STAS is consistently associated with features of aggressive tumor behavior and poor prognosis [25].

The STAS is associated with additional unfavorable prognostic markers including pleural invasion and emboli [26].

Some researchers corroborated the association between tumor budding and an unfavorable prognosis. This process has been linked to unfavorable cancer prognoses and is thought to represent epithelial-mesenchymal transition (EMT). [27] Through a series of biochemical changes, an epithelial cell, which typically interacts with the basement membrane through its basal surface, can undergo an epithelial-mesenchymal transition (EMT) and adopt the phenotype of a mesenchymal cell. The aforementioned traits include heightened invasiveness, improved migratory capacity, higher resistance to apoptosis, and markedly increased extracellular matrix component production [28]. The research by T. Taira and colleagues had similar results [29].

The findings’ reliability is diminished by widespread heterogeneity, which also limits the practicality of tumor budding in therapeutic settings. To standardize reporting on tumor budding in lung SQCC, more research is needed [30].

A higher mitotic count and the presence of tumor necrosis, fibrosis, and pleural and lymphovascular invasion are also significant pathological indicators of prognosis in lung squamous cell carcinoma [10].

Based on the histological characteristics of the peritumoral stroma, lung SQCC were classified into: “fibrous stroma type” and “thin stroma type”, according to Y. Takahashi et al. The patients with fibrous stroma-type tumors had a much worse prognosis than those with thin stroma-type cancers in terms of overall survival and recurrence-free survival [31].

Our study presents an important evolution in the approach to cancer prognosis by aiming to transcend traditional pT staging through the integration of additional pathological factors. This innovative perspective is particularly valuable in enhancing patient stratification, ultimately leading to more personalized treatment strategies tailored to individual patient needs. By developing predictive models that incorporate not only pT staging but also other relevant pathological parameters, this research holds the potential to improve prognostic accuracy significantly. Identifying new patterns and correlations can help clinicians make more informed decisions regarding treatment options, potentially leading to better overall outcomes for their patients.

Our study may have limitations (such as involving a cohort of only 99 patients), but our main goal was to highlight the significance of the morphological factors, which given their ease of evaluation, affordability, and correlation with survival should be mentioned in the pathological reports. Larger cohort analyses are required in the future.

Our research enriches the landscape of prognostic factors in SQCC, offering clinicians a nuanced understanding to optimize patient management and therapeutic outcomes. By integrating these insights into clinical practice, healthcare providers can tailor more effective treatment plans and improve patient care outcomes in this challenging disease context [32].

## 5. Conclusions

This study emphasizes the importance of specific morphological features and tumor staging in predicting outcomes for lung squamous cell carcinoma.

The multivariate analyses provide a thorough grasp of the prognostic landscape in lung squamous cell carcinoma by confirming that factors such as keratinization, neoplastic cell arrangement, tumoral budding, mitotic index, and tumor staging influence overall survival.

Our research proposes the integration of these morphological factors into a novel grading system that could fill the existing gap in routine diagnostic protocols of lung squamous cell carcinoma, which is now subjective and lacks standardization.

Future research should focus on validating these findings in larger, multi-center cohorts and exploring the molecular mechanisms underlying these histopathological characteristics. Such efforts could pave the way for more personalized therapeutic approaches and ultimately improve survival outcomes for patients with lung squamous cell carcinomas.

## Figures and Tables

**Table 1:** Overall survival on the entire cohort.

Strata	N Deaths (%)	RMST	Median Survival (95% CI)
Global	65/99 (65.65)	54.90	36.00 (23.00 to 80.00)

**Table 2:** Simple Cox regression.

Predictor	N	Death N (%)	HR (95% CI) ^1^	*p*-Value
Desmoplasia				
Severe	49	35	—	
Moderate	33	18	0.59 (0.33 to 1.05)	0.071
Mild	17	12	0.72 (0.37 to 1.38)	0.321
Grade of necrosis	98	65	1.00 (0.99 to 1.01)	0.715
Keratinization				
Yes	73	43	—	
No	26	22	2.18 (1.30 to 3.66)	0.003
Histological grading				
G1	17	9	—	
G2	50	30	1.41 (0.66 to 3.02)	0.369
G3	32	26	2.53 (1.17 to 5.47)	0.018
Location				
Right	48	34	—	
Left	51	31	0.70 (0.43 to 1.14)	0.153
Tumor’s Dimension	99	65	1.14 (1.04 to 1.24)	0.003
pT stage				
pT1	14	4	—	
pT2	42	27	3.45 (1.19 to 10.1)	0.023
pT3-pT4	43	34	4.96 (1.73 to 14.2)	0.003
pN stage				
pN0	46	29	—	
pN1	26	14	0.82 (0.43 to 1.55)	0.538
pN2	27	22	1.36 (0.78 to 2.38)	0.279
Lymphovascular invasion				
LV0	64	41	—	
LV1	35	24	1.00 (0.60 to 1.65)	0.986
Perineural invasion				
PN0	91	61	—	
PN1	8	4	0.62 (0.23 to 1.72)	0.360
Pleural invasion				
PL0 & PL1	70	41	—	
PL2 & PL3	28	2. 3	2.07 (1.23 to 3.48)	0.006
Resection margins				
R0	64	41	—	
R1	35	24	1.13 (0.68 to 1.88)	0.627
Tumoral cell distribution (tumoral center)				
Large Nests	19	6	—	
Single-cell Invasion	44	39	6.58 (2.75 to 15.7)	<0.001
Small Nests	36	20	2.46 (0.99 to 6.15)	0.053
Tumoral cell distribution (tumoral periphery)				
Large Nests	2. 3	11	—	
Single-cell Invasion	41	32	2.82 (1.41 to 5.63)	0.003
Small Nests	33	20	1.47 (0.70 to 3.07)	0.307
Tumoral Budding				
Yes	56	44	—	
No	43	21	0.37 (0.22 to 0.63)	<0.001
STAS				
Yes	49	39	—	
No	50	26	0.49 (0.30 to 0.81)	0.005
Mitotic index				
High	55	39	—	
Low	44	26	0.60 (0.37 to 0.99)	0.047
Nuclear pleomorphism				
Severe	10	7	—	
Moderate	89	58	0.76 (0.35 to 1.67)	0.491
Prominent nucleoli				
Diffuse	42	31	—	
Focal	57	34	0.66 (0.41 to 1.08)	0.101

^1^ HR = Hazard Ratio, CI = Confidence Interval, LV0 = lymphovascular invasion absent, LV1 = lymphovascular invasion present, PN0 = perineural invasion absent, PN1 = perineural invasion present, PL0 = pleural invasion absent, PL1 = pleural invasion below the internal elastic lamina, PL2 = pleural invasion beyond the external lamina elastica, PL3 = parietal pleura invasion, R0 = no residual tumor, R1 = microscopic residual tumor, and STAS = Spread Through Air Spaces.

**Table 3:** Multiple Cox regression (all predictors in the model with statistical significance).

Characteristic	N	Death N	HR (95% CI) ^1^	*p*-Value
Keratinization				
Yes	71	41	—	
No	25	21	1.53 (0.69 to 3.38)	0.292
Histological grading				
G1	17	9	—	
G2	48	28	1.64 (0.68 to 3.97)	0.270
G3	31	25	1.56 (0.54 to 4.48)	0.412
Tumor’s dimension	96	62	1.02 (0.88 to 1.18)	0.795
pT stage				
pT1	14	4	—	
pT2	41	26	2.95 (0.91 to 9.54)	0.071
pT3	41	32	2.67 (0.65 to 10.9)	0.171
Pleural invasion				
PL0 & PL1	69	40	—	
PL2 & PL3	27	22	1.06 (0.53 to 2.12)	0.877
Tumoral cell distribution (tumoral center)				
Large nests	19	6	—	
Single-cell invasion	42	37	5.31 (1.63 to 17.3)	0.005
Small nests	35	19	2.26 (0.84 to 6.12)	0.108
Tumoral cell distribution (tumoral periphery)				
Large nests	22	10	—	
Single-cell invasion	41	32	0.89 (0.33 to 2.40)	0.819
Small nests	33	20	0.84 (0.34 to 2.11)	0.714
Tumoral budding				
Yes	54	42	—	
No	42	20	0.68 (0.33 to 1.40)	0.291
STAS				
Yes	48	38	—	
No	48	24	0.74 (0.41 to 1.32)	0.304
Mitotic Index				
High	54	38	—	
Low	42	24	0.51 (0.28 to 0.94)	0.030

^1^ HR = Hazard Ratio, CI = Confidence Interval, PL0 = pleural invasion absent, PL1 = pleural invasion below the internal elastic lamina, PL2 = pleural invasion beyond the external lamina elastica, PL3 = parietal pleura invasion, and STAS = Spread Through Air Spaces.

**Table 4:** Model with all the predictors having *p*-values below 0.10.

Characteristic	N	Death N	HR (95% CI) ^1^	*p*-Value
Keratinization				
Yes	73	43	—	
No	26	22	1.81 (1.05 to 3.12)	0.032
pT stage				
pT1	14	4	—	
pT2	42	27	3.65 (1.22 to 11.0)	0.021
pT3	43	34	4.14 (1.39 to 12.3)	0.011
Tumor cell distribution (tumoral center)				
Large nests	19	6	—	
Single-cell invasion	44	39	4.27 (1.64 to 11.2)	0.003
Small nests	36	20	2.06 (0.80 to 5.29)	0.094
Tumoral budding				
Yes	56	44	—	
No	43	21	0.60 (0.33 to 1.08)	0.086
Mitotic Index				
High	55	39	—	
Low	44	26	0.53 (0.31 to 0.90)	0.019

^1^ HR = Hazard Ratio, and CI = Confidence Interval.

**Table 5:** Overall survival analysis by tumor cell keratin synthesis.

Strata Keratinization	N Deaths (%)	RMST	Median Survival (95% CI)
Yes	43/73 (58.90)	61.90	48.00 (34.00 to N/A)
No	22/26 (84.61)	35.20	19.00 (11.00 to 50.00)

**Table 6:** Overall survival analysis by pT stage.

Strata pT stage	N Deaths (%)	RMST	Median Survival (95% CI)
pT1	4/14 (28.57)	85.10	N/A
pT2	27/42 (64.28)	56.10	36.00 (20.00 to N/A)
pT3 & pT4	34/43 (79.06)	44.00	26.00 (14.00 to 64.00)

**Table 7:** Overall survival analysis by disposition of tumor cells in the central part of proliferation.

Strata Tumor Cell Distribution (Tumoral Center)	N Deaths (%)	RMST	Median Survival (95% CI)
Large nests	6/19 (31.57)	94.70	108.00 (108.00 to N/A)
Single-cell invasion	39/44 (88.63)	30.30	18.00 (11.00 to 26.00)
Small nests	20/36 (57.14)	63.90	79.00 (29.00 to N/A)

**Table 8:** Overall survival analysis by tumor budding.

Strata Tumor Budding	N Deaths (%)	RMST	Median Survival (95% CI)
Yes	44/56 (78.57)	38.90	19.00 (14.00 to 21.00)
No	21/43 (48.83)	75.50	108.00 (50.00 to N/A)

**Table 9:** Overall survival analysis by mitotic index.

Strata Mitotic Index	N Deaths (%)	RMST	Median Survival (95% CI)
High	39/55 (70.90)	44.10	23.00 (17.00 to 24.00)
Low	26/44 (59.09)	68.30	86.00 (39.00 to N/A)

## Data Availability

Data are contained within the article.
